# *Bt* proteins Cry1Ah and Cry2Ab do not affect cotton aphid *Aphis gossypii* and ladybeetle *Propylea japonica*

**DOI:** 10.1038/srep20368

**Published:** 2016-02-01

**Authors:** Yao Zhao, Shuai Zhang, Jun-Yu Luo, Chun-Yi Wang, Li-Min Lv, Xiao-Ping Wang, Jin-Jie Cui, Chao-Liang Lei

**Affiliations:** 1 Hubei Insect Resources Utilization and Sustainable Pest Management Key Laboratory, Huazhong Agricultural University, Wuhan 430070, China; 2 State Key Laboratory of Cotton Biology, Institute of Cotton Research of CAAS, Anyang 455000, China

## Abstract

Plant varieties expressing the *Bt* (*Bacillus thuringiensis*) insecticidal proteins Cry1Ah and Cry2Ab have potential commercialization prospects in China. However, their potential effects on non-target arthropods (NTAs) remain uncharacterized. The cotton aphid *Aphis gossypii* is a worldwide pest that damages various important crops. The ladybeetle *Propylea japonica* is a common and abundant natural enemy in many cropping systems in East Asia. In the present study, the effects of Cry1Ah and Cry2Ab proteins on *A. gossypii* and *P. japonica* were assessed from three aspects. First, neither of the Cry proteins affected the growth or developmental characteristics of the two test insects. Second, the expression levels of the detoxification-related genes of the two test insects did not change significantly in either Cry protein treatment. Third, neither of the Cry proteins had a favourable effect on the expression of genes associated with the amino acid metabolism of *A. gossypii* and the nutrition utilization of *P. japonica*. In conclusion, the Cry1Ah and Cry2Ab proteins do not appear to affect the cotton aphid *A. gossypii* or the ladybeetle *P. japonica*.

In the past two decades, vast plantings of insect-resistant genetically modified (IRGM) crops producing insecticidal proteins from the bacterium *Bacillus thuringiensis* (*Bt*) have contributed to the management of several major insect pests and reduced the use of insecticide sprays^1^,^2^,^3^. However, one of the risks associated with the growth of IRGM crops is their potential adverse effect on non-target arthropods (NTAs)^4^,^5^. Non-target effects assessment is one of the most important components of environmental risk assessment (ERA), which is required to authorize the IRGM crops to be released into the environment^4^,^5^.

The rapid evolution of resistance in several target pests has reduced the benefits of *Bt* crops^6^. There are usually two ways to cope with pest resistance, one is the discovery of novel cry genes, and another option is to pyramid different genes in one plant. The discovery of novel *cry* genes with new or broader activity spectra or higher toxicity is important for the development of new varieties and the management of resistance^7^. The *cry1Ah* gene is a novel insecticidal gene that was cloned from *B. thuringiensis* isolate BT8 and encodes a protein with a molecular weight of 134 kDa^7^. Although its activity spectrum and activity level are similar to those of other typical Cry1A toxins, the Cry1Ah protein is more toxic to *Helicoverpa armigera*, *Chilo suppressalis*, and *Ostrinia furnacalis* than Cry1Ac and is more toxic to *O. furnacalis* than Cry1Ab and Cry1Ie^7^. For *Plutella xylostella*, Cry1Ah exhibits toxicity similar to that of Cry1Ac, one of the most potent toxins against this pest^7^. Moreover, *cry1Ah* transgenic corn exhibited strong resistance to *O. furnacalis* larvae both in the laboratory and field^8^. Thus, high toxicity against a range of pest species makes this novel toxin a potential candidate for new *Bt* varieties. Thus a non-target effects assessment of this novel toxin should be conducted.

Another strategy to delay the evolution of pest resistance to *Bt* crops is the "pyramid" strategy, which uses plants that produce two or more toxins that kill the same pest^6^. Currently, second-generation transgenic cotton producing the *Bt* toxins Cry1Ac and Cry2Ab is the only type of *Bt* cotton grown in Australia and the predominant type of *Bt* cotton grown in India and the United States^9^. However, only first-generation *Bt* cotton producing one toxin (Cry1Ac) is grown in China, the world’s leading producer of cotton^9^. To cope with pest resistance, a new cotton cultivar (producing Cry1Ac and Cry2Ab) has been developed in China and will be commercially available in the foreseeable future^10^,^11^. The Cry2Ab protein targets various Lepidoptera pests, such as *Spodoptera exigua*, *Spodoptera frugiperda* and *Pectinophora gossypiella*^12^. Thus, non-target effect assessment before its commercial release is required.

A few studies have evaluated the effects of Cry1Ah or Cry2A proteins on non-target insects. For example, Cry1Ah corn does not appear to affect the survival, development, colony performance or behaviour of the honeybee *Apis mellifera*^13^. No significant differences were noted in the survival of *A. mellifera* or *Apis cerana* that were fed sugar syrup with Cry1Ah protein^14^. The risk assessment of Cry2A proteins have also been tested with honey bees and natural enemies. For example, the Cry2A have no acute toxicity to *A. mellifera* larvae at concentrations >10 times higher than those detected in pollen from *Bt* plants^15^. Even at concentrations higher than those expected in real-life situations, Cry2Ab does not have a detrimental effect on the green lacewing *Chrysoperla carnea* when ingested either directly or through prey^16^. When *Coleomegilla maculata* larvae were fed an artificial diet incorporated with Cry2Ab at >10-fold higher concentrations than in cotton tissue, no differences were observed in any life-table parameters in the Cry protein-containing diet treatment^17^. In addition, Li *et al.* reported that the life-table parameters of *P. japonica* were not affected when fed a rapeseed pollen-based diet containing purified Cry2A at concentrations that were >10-fold higher than in pollen^18^.

However, the potential effects of Cry1Ah and Cry2Ab on cotton aphid *A. gossypii* and ladybeetle *P. japonica* remain uncharacterized. The cotton aphid *Aphis gossypii* Glover (Homoptera: Aphididae) is a worldwide pest that damages various crops, including melon, cotton, potato, chili pepper, sweet pepper, and eggplant^19^. This pest sucks plant sap throughout the developmental period of cotton, causing seedling death, leaf curling and withering, and serious yield loss^20^. Throughout East Asia, the ladybird beetle *Propylea japonica* (Thunberg) (Coleoptera: Coccinellidae) is a common and abundant natural enemy in many cropping systems, including maize, cotton, rice, vegetables, and fruit trees^21^,^22^,^23^. Both the larvae and adults feed on aphids, thrips, spider mites, and the eggs and young larvae of Lepidoptera^24^. Furthermore, *P. japonica* can be easily reared and is amenable for testing in the laboratory, making it a suitable surrogate species for evaluating the potential effects of *Bt* proteins on predacious Coccinellidae^18^,^25^,^26^.

Most studies regarding the risk assessment of aphids and ladybird beetles have focused on biology and ecology^20^,^27^,^28^. However, the molecular response of insects to *Bt* proteins has not been elucidated. Insects have the ability to protect themselves from secondary plant metabolic and toxic chemicals and pathogens by regulating the expression of genes encoding detoxification responses^29^. Cytochrome P450 monooxygenases (P450s), glutathione *S*-transferases (GSTs) and esterases (ESTs) are three major detoxifying enzyme families that are involved in the xenobiotic metabolism of insects^30^,^31^,^32^. Mannakkara *et al.* reported that transgenic *Bt* rice expressing Cry1Ab/Cry1Ac, Cry2Aa and Cry1Ca had no marked effects on detoxification responses in *Nilaparvata lugens*^33^.

Furthermore, many study designs aimed to investigated the effects (including positive and negative effects) of *Bt* proteins on aphids and ladybeetles^20^,^27^,^28^,^34^, and here, we focused on if the effects were positive. Several key genes are involved in amino acid metabolism in pea aphids^35^, and we are interested in whether *A. gossypii* could use *Bt* proteins for amino acid metabolism. Moreover, many researchers have used the quantitative nutritional approach to investigate how food is utilized by organisms^36^,^37^. The quantitative nutritional approach consists of measuring the amount of food that is consumed, digested and assimilated, excreted, metabolized, and converted into biomass^36^. An analysis of these measurements reveals how organisms respond to different foods and which food components exert the greatest effects on growth^37^. This method has also been used to investigate how *Bt* proteins influence insects^38^,^39^,^40^.

In the present study, we investigated the effects of Cry1Ah and Cry2Ab proteins on cotton aphid *A. gossypii* and ladybeetle *P. japonica* from three aspects: (1) the toxicity of Cry proteins on the growth and developmental characteristics of *A. gossypii* and *P. japonica*; (2) the detoxification response of *A. gossypii* and *P. japonica* to Cry proteins; and (3) the effects of Cry proteins on the expression of genes that are associated with the amino acid metabolism of *A. gossypii* and the nutrition utilization of *P. japonica*. The first two aspects aim to determine whether the Cry1Ah and Cry2Ab proteins affect these two NTAs, whereas the third aspect focuses on whether the effect is positive.

## Results

### Experiments with *A. gossypii*

#### Toxicity of Cry proteins to A. gossypii

More than 90% of *A. gossypii* nymphs reached the adult stage when fed diets supplemented with Cry1Ah, Cry2Ab or pure diet, and the survival rates did not significantly differ between each Cry protein treatment and the control (*χ*^2^ test; Cry1Ah: *χ*^2^ = 0.429, *P* = 0.513; Cry2Ab: *χ*^2^ = 0.429, *P* = 0.513) (Table 1). In contrast, no nymphs developed to adults in the E-64 treatment. Moreover, no differences were noted in the nymphal development time (Mann-Whitney *U*-test; Cry1Ah: *P* = 0.963; Cry2Ab: *P* = 0.794) and adult fresh weight (Dunnett’s test; Cry1Ah: *P* = 0.077; Cry2Ab: *P* = 0.119) between each Cry protein treatment and the control (Table 1).

#### Expression of genes that are associated with detoxification responses and amino acid metabolism

**Figure 1:**
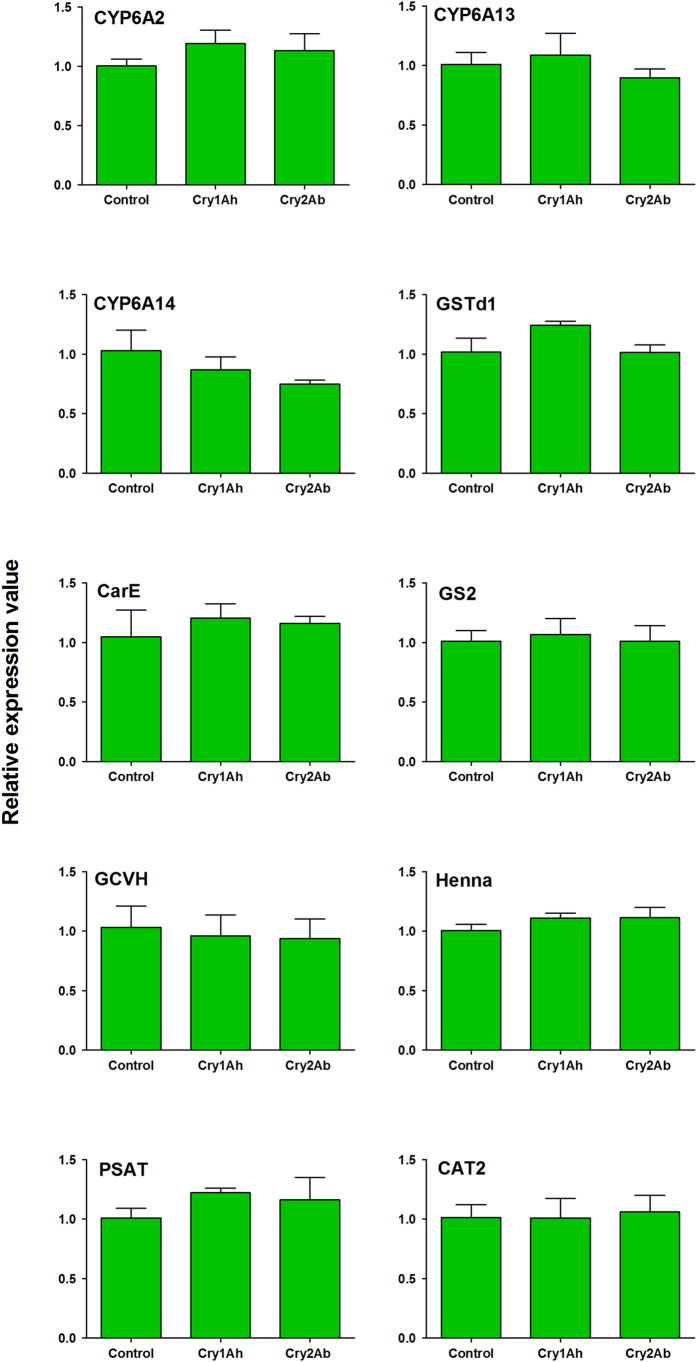
Relative expression level of detoxification and amino acid metabolism related genes of *Aphis gossypii* when fed artificial diet containing Cry1Ah or Cry2Ab. Data are means ± SE. Three replicates were tested in each treatment. Artificial diet with no *Bt* protein was served as control treatment. Means were analyzed by Tukey HSD and data yielded no significant differences at *P* < 0.05 levels.

In the Cry1Ah treatment, genes that are associated with *A. gossypii* detoxification responses [i.e., *CYP6A2, CYP6A13, glutathione S-transferase delta 1* (*GSTd1*)*, carboxylesterases* (*CarE*)] were slightly up-regulated (regulation factors did not exceed 1.3-fold) compared with the control treatment, whereas only the gene *CYP6A14* was slightly down-regulated (Fig. 1). The expression levels of genes that are related to detoxification responses also exhibited minimal change between the Cry2Ab treatment and the control (regulation factors did not exceed 1.3-fold) (Fig. 1). Similarly, the expression of genes relevant to the utilization of essential amino acids [i.e., *cationic amino acid transporter 2* (*CAT2*) and *phenylalanine hydroxylase* (*Henna*)], genes relevant to the synthesis of nonessential amino acids [i.e., *glutamine synthetase 2* (*GS2*) and *phosphoserine aminotransferase* (*PSAT*)], and a gene that is involved in the catabolism of a nonessential amino acid glycine [i.e., *glycine cleavage system h protein* (*GCVH*)], was very similar to the control in both of the Cry protein treatments (Fig. 1). In addition, no significant differences were detected in the expression levels of genes that were associated with detoxification responses and amino acid metabolism between each Cry protein treatment and the control.

### Experiments with *P. japonica*

#### Toxicity of Cry proteins to P. japonica larvae

The larval (Mann-Whitney *U*-test; Cry1Ah: *P* = 0.128; Cry2Ab: *P* = 0.660) and pupal (Cry1Ah: *P* = 0.469; Cry2Ab: *P* = 0.413) development time did not significantly differ when *P. japonica* larvae were fed a sucrose solution-based diet that contained Cry1Ah or Cry2Ab protein (Table 2). Similarly, the pupation (*χ*^2^ test; Cry1Ah: *χ*^2^ = 0.061, *P* = 0.806; Cry2Ab: *χ*^2^ = 0.015, *P* = 0.901) and eclosion (Cry1Ah: *χ*^2^ = 0.250, *P* = 0.617; Cry2Ab: *χ*^2^ = 0.143, *P* = 0.705) rates did not significantly differ between each Cry protein treatment and the control (Table 2). Moreover, no differences were found in adult fresh weight (Dunnett’s test; Cry1Ah: *P* = 0.885; Cry2Ab: *P* = 0.909) between each Cry protein treatment and the control (Table 2). In contrast, *P. japonica* in the E-64 treatment exhibited a significantly decreased larval development time (*P* < 0.001) and pupation rate (*χ*^2^ = 19.703, *P* < 0.001) compared with the control (Table 2).

#### Expression of genes that are associated with detoxification responses

**Figure 2:**
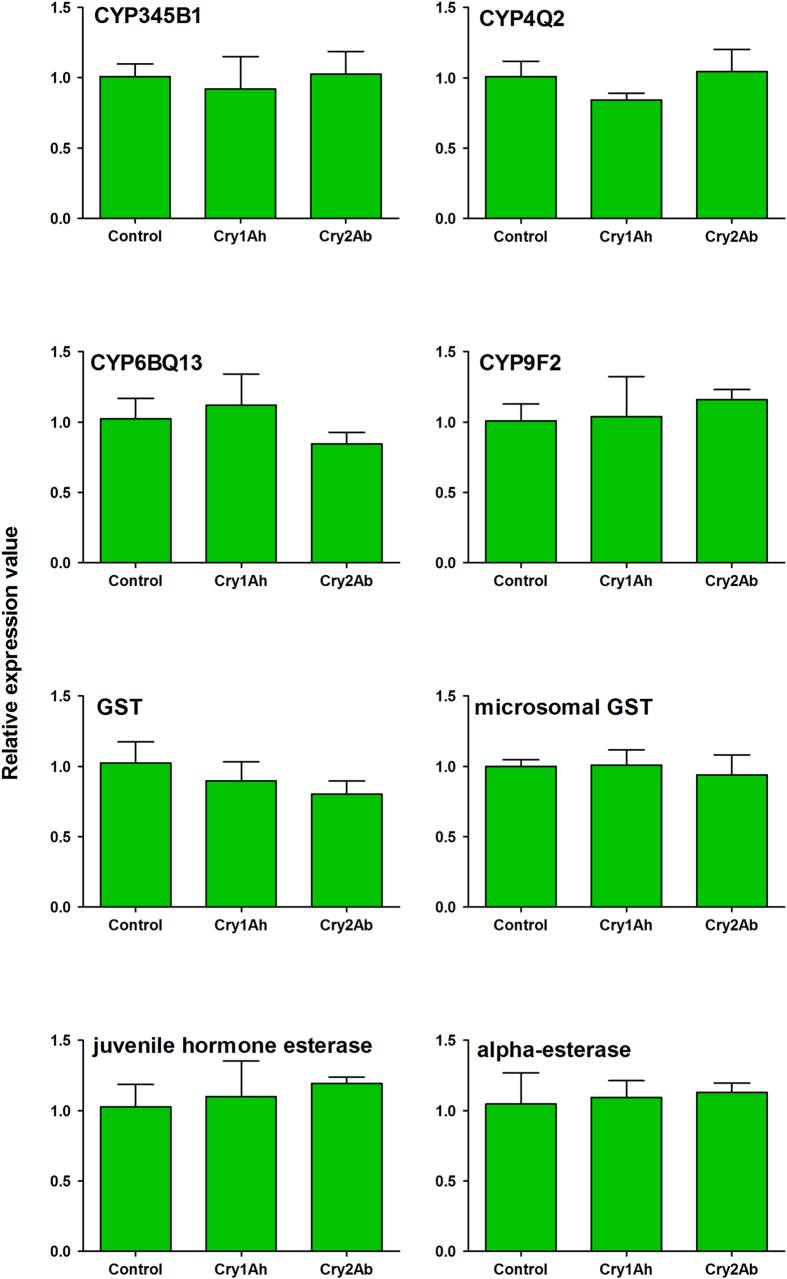
Relative expression level of detoxification related genes of *Propylea japonica* when fed sucrose solution containing Cry1Ah or Cry2Ab. Data are means ± SE. Three replicates were tested in each treatment. Sucrose solution with no *Bt* protein was served as control treatment. Means were analyzed by Tukey HSD and data yielded no significant differences at *P* < 0.05 levels.

The expression of genes that are associated with *P. japonica* detoxification responses [i.e., *CYP345B1, CYP4Q2, CYP6BQ13, CYP9F2, GST, microsomal GST*, *juvenile hormone esterase*, *alpha-esterase*] were slightly altered (regulation factors did not exceed 1.2-fold) in each Cry protein treatment compared with the control (Fig. 2), and the alterations were not significantly different.

#### Nutrition utilization of P. japonica

The ELISA results showed that the concentrations of Cry2Ab was 458.04 ± 12.43 μg/g fresh weight (FW) in the fresh diets and 436.71 ± 13.48 μg/g FW in the diets that had been stored at 4 °C for 24h. The difference was not significant between this two diets (*t* = 1.163, *df* = 4, *P* = 0.309). The relative growth rate (RGR) (least significant difference (LSD) test; Cry1Ah: *P* = 0.816; Cry2Ab: *P* = 0.149), efficiency of conversion of ingested materials (ECI) (Cry1Ah: *P* = 0.554; Cry2Ab: *P* = 0.072), efficiency of conversion of digested food (ECD) (Cry1Ah: *P* = 0.748; Cry2Ab: *P* = 0.085) and efficiency of approximate digestibility (EAD) (Cry1Ah: *P* = 0.857; Cry2Ab: *P* = 0.388) did not significantly differ when *P. japonica* larvae were fed a pork-liver-based artificial diet that contained Cry1Ah or Cry2Ab protein (Table 3). No significant difference was detected in relative consumption rates (RCR) between the Cry1Ah treatment and the control (*P* = 0.333), but the RCR was significantly lower in the Cry2Ab treatment than in the control treatment (*P* = 0.038) (Table 3).

## Discussion

Non-target risk assessment for transgenic crops should be case specific, depending on the plant, transgene, and intended release environment^4^. In the present study, we focused on the novel insecticidal protein Cry1Ah and a toxin with prospects for commercial release in China, Cry2Ab. The concentrations of Cry1Ah and Cry2Ab used in our toxicity bioassays were at least 10 times higher than that measured in *Bt* cotton pollen^11^. This high concentration represents a worst-case exposure scenario and could increase the certainty of the hazard assessment^4^. The bioassay results revealed that neither of the Cry proteins affected the growth or developmental characteristics of the two tested insects. Moreover, the expression of the detoxification-related genes of the two tested insects was not significantly altered in either of the Cry proteins treatments. Our results indicate that neither Cry protein has a favourable effect on the expression of genes that are associated with the amino acid metabolism of *A. gossypii* and the nutrition utilization of *P. japonica*.

A number of studies aimed to quantify the Cry protein concentration in aphids that have fed *Bt*-transgenic plants. In the majority of studies, Cry proteins were either absent or detected at very low levels that might have been due to contamination^41^. However, Burgio *et al.* repeated experiments with a pre-flowering stage of Cry1Ac-expressing oilseed rape in a growth chamber and again detected Cry1Ac protein uptake by *Myzus persicae*^42^. These results confirm the findings of their previous study and highlight the potential for Cry protein uptake by aphids^42^. In our previous study, a small fraction of Cry1Ac proteins were detected in *A. gossypii* feeding on *Bt* cotton cultivar^10^. Because the aphids were previously checked under a microscope, it is unlikely that the samples were contaminated^10^. Moreover, Cry1Ac and Cry3Aa bind to the aphid gut epithelium and exhibited low toxicity against the pea aphid *Acyrthosiphon pisum*^43^. Thus, we believe that Cry proteins may potentially affect aphids. In present study, the results suggest that Cry1Ah and Cry2Ab have no impact on the performance of *A. gossypii*. Similarly, *Bt* (Cry1Ac) cotton did not have any influence on the performance of *A. gossypii*^27^. In contrast, *A. gossypii* on *Bt* (Cry1A) + *CpTI* cotton exhibited reduced survival rates and an earlier occurrence of peak daily mortality in the first generation^20^.

In a previous study, we evaluated the effects of *Bt* (Cry1Ac + Cry2Ab) cotton on *P. japonica* through its prey *A. gossypii*. Development was delayed, and the pre-oviposition period was significantly longer when *P. japonica* was fed *A. gossypii* reared on *Bt* cotton^10^. Poor prey quality may account for the negative effects because other studies have indicated that plant allelochemicals can affect the nutritional suitability of herbivores, with potential effects on the performance of predators at the third trophic level of the food chain^44^. In the present study, a 2 M sucrose solution was used as carrier to deliver Cry proteins to *P. japonica* because this method worked in previous studies^28^,^45^, and this technique may avoid the problems discussed above. Our data revealed no distinct differences in any of the *P. japonica* life-table parameters between the Cry protein treatments and the control. The results are consistent with those of numerous other studies that were conducted to assess the effect of Cry proteins on *P. japonica*. The life-table parameters of *P. japonica* were not affected when fed a rapeseed pollen-based diet containing purified Cry1C or Cry2A at concentrations that were >10-fold higher than in pollen^18^. Cry proteins Cry1Ab, Cry1Ac, and Cry1F have no direct toxicity to *P. japonica*^46^. A tritrophic study also confirmed that the Cry1Ac protein is unlikely to have detrimental effects on this representative species^24^.

To date, little is known about the detoxification response of non-target insects to *Bt* proteins. In our study, five detoxification-related genes of *A. gossypii* were obtained from the transcriptome data^47^, and eight genes that are involved in the *P. japonica* detoxification response were obtained from Tang *et al.*^48^. Tang *et al.* analysed the transcriptome of an insecticide-resistant *P. japonica* strain and identified potential candidate genes for conferring insecticide resistance, including P450s, GSTs and ESTs^48^. Previous studies have suggested that *CYP6s* are associated with insecticide resistance^49^,^50^. For example, the overexpression of *CYP6BQ9* in *Tribolium castaneum* brain tissue was partly responsible for the deltamethrin resistance^49^, and the overexpression of *CYP6G1* in *Drosophila melanogaster* caused resistance to dithiothreitol (DDT) and imidacloprid^50^. *CYP340s* with a relatively high expression in the midgut probably contribute to the detoxification of insecticides or plant toxins in *P. xylostella*^51^. *CYP9s* also have been indicated in relation to the response to plant allelochemicals and xenobiotics^52^. In our study, *CYP6A2, CYP6A13,* and *CYP6A14* of *A. gossypii* and *CYP345B1, CYP4Q2, CYP6BQ13,* and *CYP9F2* of *P. japonica* did not significantly respond to Cry1Ah and Cry2Ab. Similar results were noted for *CYP6AY1* and *CYP4CE1* of *N. lugens* reared on transgenic rice^33^. Another study found that the expression of *CYP6AE14*, *CYP6B2* and *CYP9A12* was suppressed in *H. armigera* larvae that fed the Cry1Ab toxin, which might be related to the low susceptibility of the species to Cry1Ab toxin^53^.

Similarly to P450s, GSTs and ESTs can function broadly in xenobiotic detoxification^31^,^32^. The *delta* GST classes were uniquely identified in insects and have been implicated in insecticide resistance^31^. CarE is involved in organophosphorus insecticide resistance and the metabolism of xenobiotic compounds in numerous insect species^54^. Our data revealed that *GSTd1* and *CarE* of *A. gossypii* and *GST*, *microsomal GST*, *juvenile hormone esterase* and *alpha-esterase* of *P. japonica* were not significantly regulated by Cry1Ah and Cry2Ab. Likewise, *GST* did not exhibit any significant difference when *N. lugens* was reared on transgenic rice^33^. In total, our results suggest that the Cry1Ah and Cry2Ab proteins have no distinct effects on the detoxification-related gene regulation of *A. gossypii* and *P. japonica*.

Our results demonstrate that the genes of *A. gossypii* that are relevant to the utilization of essential amino acids (*CAT2* and *Henna*) and the synthesis of nonessential amino acids (*GS2* and *PSAT*) as well as a gene that is involved in the catabolism of a nonessential amino acid (*GCVH*) did not distinctly respond to the Cry1Ah and Cry2Ab proteins. Elevated CO_2_ up-regulated the expression of genes that are relevant to amino acid metabolism in the aphid *A. pisum*, indicating that pea aphids manipulate their amino acid metabolism to favour the population growth of the aphid under elevated CO_2_^55^. However, similar results were not observed in our study, indicating that the amino acid metabolism of *A. gossypii* was not altered by either of the Cry proteins.

Nutritional ecology is central to proper interpretations of life history phenomena (e.g., manner of feeding, habitat selection, defence, and reproduction), both in ecological and evolutionary time^37^. Many studies of nutritional ecology have focused on target pests^38^,^39^,^40^. When *H. armigera* were fed transgenic *Bt* (Cry1Ac) cotton, the RGR, ECI and ECD were significantly reduced^38^. This is not surprising, given that *H. armigera* is sensitive to Cry1Ac. Similarly, the *Bt* (Cry1Ac) cotton significantly reduced the relative growth, consumption, metabolic rates and other nutritional indices of *S. frugiperda*^39^. In contrast, no significant difference was detected for any nutritional indices of *O. furnacalis* and *H. armigera* when fed phytase transgenic maize^40^. In our study, the RGR, ECI, ECD and EAD indices of *P. japonica* were not significantly altered in the *Bt* treatment. However, the RCR of the Cry1Ah treatment was reduced by 7%, and a marginally significant difference (*P* = 0.038) was observed compared with the control. We believe that this decrease is minor and is unlikely to affect this species in the field given that the *Bt* concentration that was used in our study is considerably higher than that noted under field conditions.

In summary, the current study suggests that Cry1Ah and Cry2Ab proteins have no adverse or beneficial effect on the cotton aphid *A. gossypii* or the ladybeetle *P. japonica*. Because these two *Bt* toxins have not been promoted in China, the present study is valuable for decision-making in the commercialization of new *Bt* varieties and in the establishment of appropriate integrated pest management strategies in the field.

## Methods

### Insects

Ladybeetles *P. japonica* were collected in April 2014, and cotton aphids *A. gossypii* were collected in July 2013 at the experimental field station of the Institute of Cotton Research, Chinese Academy of Agricultural Sciences (CAAS). *P. japonica* was maintained on pea aphids *Acyrthosiphon pisum* over 4 generations, and a colony of *A. gossypii* was maintained on a non-transgenic cotton cultivar. Both insects were reared at 25 ± 1 °C and 75 ± 5% relative humidity (RH) under a16:8 h light:dark (L:D) cycle.

### Insecticidal compounds

The insecticidal compounds that were used in this study include the protease inhibitor E-64 [N-[N-(L-3-trans-carboxyoxirane-2-carbonyl)-L-leucyl]-agmatine)] and the *Bt* proteins Cry1Ah and Cry2Ab. E-64 was purchased from Sigma-Aldrich (St. Louis, MO, USA). Activated Cry1Ah and Cry2Ab proteins were supplied by the Biotechnology Research Laboratory, Institute of Plant Protection, Chinese Academy of Agricultural Sciences. The Cry1Ah and Cry2Ab protoxins from *Bacillus thuringiensis* had been expressed as single-gene products in *Escherichia coli*. The *E. coli*-expressed protoxin inclusion bodies then were dissolved and trypsinized. Subsequently they were isolated and purified by ion exchange HPLC followed by the desalting and lyophilization of the pure fractions. A bioassay showed that the LC_50_ (concentration resulting 50% mortality) of our batch of Cry1Ah and Cry2Ab proteins were 7 μg/ml and 10 μg/ml diet when neonates of *H. armigera* were fed a *Bt*-containing artificial diet for one week.

### Experiments with *A. gossypii*

#### Toxicity of Bt proteins to A. gossypii

Membrane feeding assays^43^ were used to assess the toxicity of Cry1Ah and Cry2Ab against *A. gossypii*. The artificial diet was the diet A5 reported by Febvay *et al.*^56^, with a sucrose content changed to 25%. Glass tubes (cylindrical glass tubes, 25 mm diameter, and 50 mm height) with openings at both ends were used as feeding containers. One end of the glass tube was covered with a stretched Parafilm sachet containing 200 μl of artificial diet. Aphids were introduced into the tube from the other end, which was then covered with a fine mesh.

Fifteen 2^nd^ instar (1^st^ instar aphids were not able to penetrate through the stretched Parafilm) aphids that were grown on non-transgenic cotton were transferred to the artificial diet device, with five replicates for each treatment. The aphids were fed the artificial diet containing (1) 500 μg/ml Cry1Ah; (2) 500 μg/ml Cry2Ab; (3) 600 μg/ml E-64 (a highly specific cysteine proteinase inhibitor^57^; positive control); or (4) no added toxin (negative control). The diet was replaced every two days, and assays were conducted for a period of 7 days. The assays were conducted in a growth chamber at 24 ± 1 °C and 75 ± 5% RH under a 16:8 L:D cycle. Aphids were observed twice per day (9:00 am and 9:00 pm), and their development and mortality were recorded. When the adults emerged, ten randomly selected aphids were weighed (within 12 h) for each replicate.

#### Expression of genes that are associated with detoxification responses and amino acid metabolism

*A. gossypii* were reared on artificial diets that did or did not include *Bt* proteins as described above. When the adults emerged, aphids were frozen in liquid nitrogen and stored at –80 °C for further use. The entire bodies of three pools of ten individuals from each sample were used for total RNA extraction. Total RNA was extracted by the SV Total Isolation System (Promega, Madison, WI, USA) following the manufacturer’s instructions. The concentration and quality of total RNA were determined using a NanoDrop 2000c spectrophotometer (Thermo Fisher Scientific, USA). The first-strand cDNA of each sample was synthesized from 1 μg of total RNA using a PrimeScript RT reagent kit with gDNA eraser (Perfect Real Time) (TaKaRa Biotechnology (Dalian) Co., Ltd.).

Gene functions were determined by searching BLASTX and Kyoto Encyclopedia of Genes and Genomes with the cotton aphid transcriptome data^47^. To confirm the transcriptome assemblies, the PCR products of all of the putative genes were sequenced. Quantitative real-time PCR (qPCR) was performed using the Mastercycler ep realplex system (Eppendorf, Hamburg, Germany). Gene-specific primers (Supplementary Table S1) were designed using Beacon Designer 7.6 and synthesized by GENEWIZ Co. Ltd. (Beijing, China). Dimethyladenosine transferase (GenBank: KF018923) and peptidyl-prolyl *cis-trans* isomerase (GenBank: KF018924) were used as endogenous controls^47^. The reaction was performed as follows: 2 min at 95 °C followed by 40 cycles at 95 °C for 15 s, 58 °C for 30 s and 72 °C for 30 s. A melting curve was used to detect a single gene-specific peak and the absence of primer dimer peaks. GoTaq qPCR Master Mix (Promega, Madison, WI, USA) was used to measure the mRNA levels according to the manufacturer’s instructions. A fivefold dilution series was used to construct a relative standard curve to determine the PCR efficiencies and for quantification analysis. Each reaction was performed in triplicate (technical repeat) with three independent biological replicates. The transcript levels were calculated by the comparative 2^−△△CT^ method^58^.

### Experiments for *P. japonica*

#### Toxicity of Bt proteins to P. japonica larvae

A bioassay was conducted to test the toxicity of Cry1Ah and Cry2Ab to *P. japonica* as described by Alvarez-Alfageme *et al.*^45^. On the first day of each instar, *P. japonica* larvae were individually fed with a 2 M sucrose solution containing (1) 500 μg/ml Cry1Ah; (2) 500 μg/ml Cry2Ab; (3) 400 μg/ml E-64 (positive control); or (4) no added toxin (negative control). After 24 h, larvae were individually transferred to a clean 7-ml centrifuge tube that was sealed with cotton gauze and subsequently fed *ad libitum* with pea aphids until the next moult. Thirty-five individual *P. japonica* larvae were tested for each treatment. Larval development and mortality were recorded twice per day (9:00 am and 9:00 pm), and emerging adults were sexed and weighed (within 12 h).

#### Expression of genes that are associated with detoxification responses

Newly hatched *P. japonica* larvae were reared on *A. pisum* until the 3^rd^ instar. On the first day of the 4^th^ instar, *P. japonica* larvae were individually fed the 2 M sucrose solution containing Cry1Ah, Cry2Ab or no added toxins. After 24 h, larvae were frozen in liquid nitrogen and stored at –80 °C for further use. The entire bodies of three pools of five individuals from each sample were used for total RNA extraction. RNA extraction, first-strand cDNA synthesis and qPCR were performed as described above. Gene-specific primers (Supplementary Table S2) were from Tang *et al.*^48^, and *β-actin* was used as a reference gene.

#### Nutrition utilization of P. japonica

Many previous studies have used *P. japonica* artificial diets containing saccharides, insect tissue and viscera of livestock^59^,^60^. An artificial diet that was optimized previously^60^ was used in the present study. This artificial diet includes fresh pork liver + pure honey + sucrose (regulated weight ratio of 5:1:1, respectively) and 0.3% (to the total weight of the diet) olive oil. Fresh pork liver was mashed in a blender with pure honey, sucrose and olive oil in the described proportions. Cry1Ah and Cry2Ab were mixed with the artificial diet respectively at final concentrations of 500 μg/ml. An artificial diet with no *Bt* protein was used as a control. The artificial diet of each treatment was kept in a plastic jar that was covered by Parafilm and stored at 4 °C. Every other day, another batch of pork-liver-based artificial diets (with or without Cry proteins) was prepared to keep the diets fresh and to avoid the degradation of the Cry proteins. The methods of measuring the stability of the Cry2Ab protein in the artificial diets are provided in the Supplementary Information.

The 4^th^ instar larvae (within 12 h) were tested in this assay. Before this assay, samples of artificial diets (n = 50) and 4^th^ instar larvae (n = 50) were oven-dried at 80 °C for 72 h to calculate the proportion of dry matter and water content^36^,^38^. Every day, approximately 60 μl of artificial diet was placed on a square of Parafilm (length of a side = 20 mm) and offered to each larva, which was reared in a 7-ml centrifuge tube sealed with cotton gauze. After 24 h, the remaining artificial diets and the frass that was produced by each larva were collected and oven-dried as above. The development time of each 4^th^ instar larva was calculated from the beginning of 4^th^ instar to pre-pupa. The pre-pupa were oven-dried as above, and the dry weights were determined. Thirty-five larvae were reared individually for each treatment.

The nutrition utilization indices, including the RGR, RCR, ECI, ECD, and EAD, were determined gravimetrically following the methods of Waldbauer (1968)^36^ and Scriber & Slansky (1981)^37^. The amount of artificial diet (mg) that was ingested, the amount of frass that was produced and the larval body weight were calculated as dry weights.

#### Data analyses

The details of the data analyses are provided in the Supplementary Information.

## Additional Information

**How to cite this article**: Zhao, Y. *et al.* *Bt* proteins Cry1Ah and Cry2Ab do not affect cotton aphid *Aphis gossypii* and ladybeetle *Propylea japonica*. *Sci. Rep.* **6**, 20368; doi: 10.1038/srep20368 (2016).

## Supplementary Material

Supplementary Information

## Figures and Tables

**Table 1:** Life-table parameters of *Aphis gossypii* when fed artificial diet containing Cry1Ah, Cry2Ab or E-64.

Treatment	Percent nymphs developed to adults^a^	Days to adult^b^	Adult fresh weight (mg)^c^
Control: diet only	94.67 ± 2.49	5.57 ± 0.06	0.62 ± 0.02
Cry1Ah 500 μg/ml diet	93.33 ± 2.98	5.58 ± 0.06	0.57 ± 0.02
Cry2Ab 500 μg/ml diet	92.00 ± 2.49	5.60 ± 0.06	0.57 ± 0.02
E-64 600 μg/ml diet	0	–	–

Data are means ± SE. Each toxin treatment was compared to the control. Each treatment has five replicates. Fifteen 2^nd^ instar nymphs (within 12 h) were tested for each replicate. "-" indicates no individual reached the adult stage in E-64 treatment.

^a^*χ*^*2*^ test.

^b^Mann–Whitney *U* test.

^c^Dunnett’s test.

**Table 2:** Life-table parameters of *Propylea japonica* when fed a sucrose solution-based diet containing Cry1Ah, Cry2Ab or E-64.

Treatment	Larval development time (d)^a^	Pupation rate (%)^b^	Pupal development time (d)^a^	Eclosion rate (%)^b^	Adult fresh weight (mg)^c^	
					Female	Male
Control: pure diet	11.63 ± 0.14	91.43	4.25 ± 0.04	93.75	5.1 ± 0.18	4.42 ± 0.15
Cry1Ah 500 μg/ml diet	11.24 ± 0.18	97.14	4.15 ± 0.13	100	5.25 ± 0.18	4.25 ± 0.16
Cry2Ab 500 μg/ml diet	11.55 ± 0.20	94.29	4.11 ± 0.11	100	5.24 ± 0.15	4.52 ± 0.14
E-64 400 μg/ml diet	15.9 ± 0.51*	14.29*	4.4 ± 0.19	100	4.87 ± 0.26	4.18

Data are means ± SE. Each toxin treatment was compared to the control. An asterisk denotes a significant difference between a toxin treatment and the control. Thirty-five larvae were tested for each treatment. Sample of male fresh weight in E-64 treatment was not enough for data analyze.

^a^Mann–Whitney *U* test.

^b^*χ*^*2*^ test.

^c^Dunnett’s test.

**Table 3:** Nutrition utilization of *Propylea japonica* when fed artificial diet containing Cry1Ah or Cry2Ab protein.

Treatment	Nutrition utilization indices				
	RGR(mg/mg/day)^a^	RCR(mg/mg/day)^a^	ECI(%)^a^	ECD(%)^a^	EAD(%)^a^
Control: pure diet	0.12	0.66 ± 0.03	18.95 ± 1.05	22.14 ± 1.34	86.42 ± 0.81
Cry1Ah 500 μg/ml diet	0.11	0.59 ± 0.03	20.02 ± 0.91	23.51 ± 1.17	85.78 ± 0.85
Cry2Ab 500 μg/ml diet	0.13 ± 0.01	0.61 ± 0.02*	21.19 ± 0.77	25.03 ± 1.02	85.34 ± 1.08

Data are means ± SE. Each toxin treatment was compared to the control. An asterisk denotes a significant difference between a toxin treatment and the control. Thirty-five larvae were tested for each treatment.

^a^ANCOVA followed by LSD test.
