# Supplementary material for: Bt proteins Cry1Ah and Cry2Ab do not affect cotton aphid Aphis gossypii and ladybeetle Propylea japonica
Source: Sci Rep. 2016 Feb 1;6:20368. doi: 10.1038/srep20368 (PMC4734323; doi:10.1038/srep20368)
Supplement: Supplementary Information [file srep20368-s1.pdf]

Supplementary Information

Bt proteins Cry1Ah and Cry2Ab do not affect cotton aphid *Aphis gossypii* and ladybeetle *Propylea japonica*

Yao Zhao[1,2], Shuai Zhang[2], Jun-Yu Luo[2], Chun-Yi Wang[2], Li-Min Lv[2], Xiao-Ping Wang[1], Jin-Jie Cui[2,*] & Chao-Liang Lei[1,*]

[1]Hubei Insect Resources Utilization and Sustainable Pest Management Key Laboratory, Huazhong Agricultural University, Wuhan 430070, China, [2]State Key Laboratory of Cotton Biology, Institute of Cotton Research of CAAS, Anyang 455000, China.

*Correspondence and requests for materials should be addressed to C.-L.L. (ioir@mail.hzau.edu.cn) or J.-J.C. (cuijinjie@126.com)

This Supplementary Information contains:

Supplementary Methods

Supplementary Tables S1-S2

Supplementary Methods

The stability of the Cry2Ab protein in the artificial diets. The stability of the Cry2Ab protein in the artificial diets was measured with an enzyme-linked immuno-sorbent assay (ELISA) using the Cry2Ab detection kit (EnviroLogix Inc., Portland, ME, USA). The samples for ELISA measurements were collected from: 1) fresh diets, 2) diets that had been stored at 4°C for 24h and provided to *P. japonica* next day. For Cry protein extraction, samples of artificial diets were weighed and homogenized with PBST buffer at a ratio of 1:200 (sample [mg]: buffer [µl]). After centrifugation and appropriate dilution of the supernatants, ELISA was performed according to the manufacturer's instructions. The optical density (OD) values were read with a microplate spectrophotometer (Synergy 2, BioTek, USA). The measured OD values were calibrated to a range of Cry2Ab concentrations made from purified protein solution.

Data analyses. Pair-wise statistical comparisons were made between the Cry protein or E-64 treatment and the control treatment. Data analyzed with ANOVA were first checked whether they satisfy the assumptions for parametric analyses (normal distribution of residuals and homogeneity of error variances). One-way ANOVA and Dunnett's tests were used to analyse the weights of newly emerged adults of aphids and ladybeetles. Mann–Whitney *U* tests were used to compare the developmental periods, and χ^2 tests were used to compare the mortality rates because such data did not satisfy the assumptions for parametric analyses. Student's *t*-tests were conducted to compare Cry protein concentrations in samples from: fresh diets; diets that had been stored at 4°C for 24h and provided to *P. japonica* next day. One-way ANOVA and Tukey's Honestly Significant Difference (HSD) tests were performed to analyse the qRT-PCR data.

The nutrition utilization data for *P. japonica* were analysed using an analysis of covariance (ANCOVA) with the initial weight as a covariate for RCR and RGR, whereas food consumption was a covariate for ECI and AD to correct for the effect of variation on the growth and food assimilated on intake and growth[61]. Food assimilation [i.e., artificial diets ingested (mg) - frass produced (mg)] was also used as a covariate to analyse the ECD parameter[62]. The assumption of a parallel slope between covariate and dependent variable was satisfied for each analysis. Means were separated using LSD tests.

All of the statistical analyses were conducted with SPSS 17.0 (SPSS Inc., Chicago, IL, USA.).

References

61. Raubenheimer, D. & Simpson, S. Analysis of covariance: an alternative to nutritional indices. *Entomol. Exp. Appl.* **62**, 221-231 (1992).

62. Hägele, B. F. & Rowell-Rahier, M. Dietary mixing in three generalist herbivores: nutrient complementation or toxin dilution? *Oecologia* **119**, 521-533 (1999).

Supplementary Table S1 Primers sequences used for qRT-PCR of *A. gossypii*

Gene name	Accession number	Primer sequence (5'-3')
dimethyladenosine transferase[a]	KF018923	Sense: CCAAGCCAGGAGACAAACTAT
		Antisense: CGAGGTTCTATACGCACTACATT
peptidyl-prolyl cis-trans isomerase[a]	KF018924	Sense: ATTGAAACTCGCCCAGATGT
		Antisense: ATCACCAGTAATCACAGATTCTCC
cytochrome P450 6a2[a]	KR028422	Sense: GGAGAACGATGCGACAGAA
		Antisense: TTCAGACCGAAAGCCGATG
cytochrome P450 6a13[a]	KR028423	Sense: AATTGTTGATGTGAGTGAAGCAG
		Antisense: TACGACGAAATTCTGAATCTGGAT
cytochrome P450 6a14[a]	KR028424	Sense: GTACGACGGTCACAGAGAAC
		Antisense: CTCCACATCCTTGTAACATTCCTA
glutathione *S*-transferase delta 1[a]	KR028425	Sense: GCAATCATCGTGTACTTGGTTC
		Antisense: TTTCGGCACTCCAGCAAA
carboxylesterases[b]	AY485218	Sense: CATACCCTACGCTCAACCAC
		Antisense: GCAATCTTCACTTCCAACGA
glutamine synthetase 2[a]	KR028426	Sense: AAGAGTTTGGCGTTGTGGTA
		Antisense: GCTTGAGTTGAGAAATTACAGTGG
glycine cleavage system h protein[a]	KR028427	Sense: TATGCTGAGCTACCTTCTGTTG
		Antisense: TTTATCAATGCTGGTCTTGTTTCC
phenylalanine hydroxylase[a]	KR028428	Sense: TGTTGGTGGGCTTCTTTCTT
		Antisense: CTGTGATGTCTGATGTACTGTGT
phosphoserine aminotransferase[a]	KR028429	Sense: GGAGGTCTTGTTGCTATGGAA
		Antisense: AATGGCACGGTTATACGACTT
cationic amino acid transporter 2[a]	KR028430	Sense: CGCCAGGAGGATTCAGTG
		Antisense: GACACGAACACCAGCAGTA

[a]Primers cited from this study.

[b]Primers cited from Gong et al., 2014[54].

Supplementary Table S2 Primers sequences used for qRT-PCR of *P. japonica*

Gene name	Primer sequence (5’-3')
β-actin[a]	Sense: GTTACTCTTTCACCACCACA
	Antisense: GGGCAACGGAATCTTT
cytochrome P450 CYP345B1[a]	Sense: TGCCATACAGACGACTTG
	Antisense: TCCTTCATTGTCCTCCA
cytochrome P450 CYP4Q2[a]	Sense: ATTTGCGAAGAGGTTGA
	Antisense: CGTAAGATGGGTGTTCAT
cytochrome P450 CYP6BQ13[a]	Sense: TTCTTAGCGGGTTTCG
	Antisense: ATGGCTTCATAAGTAATCCTAC
cytochrome P450 CYP9F2[a]	Sense: AGAGCCCAACTATGAAGC
	Antisense: GATATGCCCACGGAGC
glutathione *S*-transferase[a]	Sense: ACCTCAAAGACCCTTATTACC
	Antisense: TCTCCACAGCCCGAAA
microsomal glutathione s-transferase[a]	Sense: TTTGACTGAACCCAGCGTAG
	Antisense: GGCGAAACCAATCGTATGT
juvenile hormone esterase[a]	Sense: GATGCCAGTTTCCCTAT
	Antisense: TTTCTATCTTTCCCTCCA
alpha-esterase[a]	Sense: CAGGTGGAGCGTCAGTT
	Antisense: TTGGGCTATTGCTTTGTG

[a]Primers cited from Tang et al., 2014[48].
